# Maternal syphilis in the Federal District, Brazil: a five-year analysis of notified data (2019–2023)

**DOI:** 10.3389/fepid.2025.1613872

**Published:** 2025-11-21

**Authors:** Paula Beatriz de Medeiros Santiago, Maria Eduarda Santiago Meneses, Luiza de Lima Pereira, Maria Fernanda Santiago Meneses, Pamela Araújo da Silva, Fabiana Nunes de Carvalho Mariz, Ciro Martins Gomes, Carla Nunes de Araújo

**Affiliations:** 1 Laboratório de Interação Patógeno-Hospedeiro (LIPH), Instituto de Ciências Biológicas, Universidade de Brasília, Brasília, DF, Brazil; 2 Faculty of Medicine, Centro Universitário Unieuro, Brasília, DF, Brazil; 3 Programa de Pós-Graduação em Ciências Médicas, Universidade de Brasília, Brasília, DF, Brazil; 4 Universidade Católica de Brasília, Brasília, DF, Brazil; 5 Faculdade de Ciências e Tecnologias em Saúde, Campus Ceilândia, Universidade de Brasília, Brasília, DF, Brazil

**Keywords:** maternal syphilis, socioeconomic factors, demographic profiles, health inequalities, Brazil

## Abstract

Syphilis, caused by the bacterium *Treponema pallidum*, is among the most prevalent STIs globally and represents a significant public health challenge in Brazil. Vertical transmission can occur at any stage of pregnancy, leading to severe consequences such as miscarriage, stillbirth, preterm birth, and low birth weight. In severe cases, congenital syphilis may result, potentially causing deformities, neurological damage, or even neonatal death. Maternal syphilis (MS) occurrence is often influenced by social inequalities, meager income, and educational levels, which present further public health challenges. We evaluated the incidence of maternal syphilis in the Administrative Regions of the Federal District (FD), Brazil, from 2019 to 2023, highlighting the distribution of notified cases regarding the sociodemographic profile of affected pregnant women. We utilized data from the *Info Saúde DF* portal (https://info.saude.df.gov.br/sifilissalasit/) and the latest report from the most recent District Household Sample Survey. The cumulative incidence of MS was 25.3 cases per 1,000 live births. Simple linear regression analysis was used to assess the association between MS incidence and sociodemographic characteristics. A moderate negative correlation was observed (*r* = −0.4038), indicating that higher education levels are associated with a lower incidence of MS. Lower-income populations living in the FD, Brazil, face a heightened risk of maternal syphilis, possibly due to a combination of economic and social factors, suggesting that public health policies aimed at controlling the disease should prioritize this population.

## 1 Introduction

Syphilis is a systemic, chronic, and curable disease, caused by the bacterium *Treponema pallidum*, that affects only humans. Its clinical manifestations arise from local inflammatory responses induced by the replication of the spirochetes within tissues. Most individuals with syphilis are asymptomatic, and even when symptoms are present, they often go unnoticed, facilitating transmission. Disease progression follows primary, secondary, and tertiary stages over a period of more than ten years. Primary syphilis presents as a painless ulcer, secondary syphilis as skin rashes and systemic symptoms, and tertiary syphilis as severe damage to organs like the heart and nervous system (1, 2). The disease includes cycles of active and latent phases; without treatment, it has the potential for systemic complications (2).

In addition to direct sexual transmission, syphilis can be transmitted from mother to fetus during pregnancy or delivery if the mother is inadequately treated (2). Maternal syphilis (MS) is concerning as *T. pallidum* can cross the placenta at any stage of pregnancy (3). When left untreated or improperly managed, MS can have devastating consequences for the fetus, including miscarriage, preterm birth, stillbirth, and neonatal death. It is estimated that around 30% of pregnancies involving untreated MS result in fetal death, either *in utero*, as stillbirth, or shortly after delivery (1).

Syphilis remains one of the most prevalent STIs worldwide, with an estimated 6 million new cases each year (4). Since the early 2000s, several countries have reported outbreaks of syphilis, which are linked to a rise in unsafe sexual practices, possibly influenced by the perception of reduced risk due to advances in HIV treatment (5–8). In Brazil, the disease is a significant public health issue. According to the Brazilian Ministry of Health, in 2022, the country reported 99.2 cases of acquired syphilis per 100,000 inhabitants, 32.4 cases per 1,000 live births of MS, and 10.3 cases of congenital syphilis (CS) per 1,000 live births. The Federal District (FD) reported an incidence of 28.9 MS and 11.6 CS per 1,000 live births, values that exceed the national averages (9).

Beyond its direct impact on maternal and child health, MS also serves as an indicator of broader public health challenges, often rooted in social inequalities such as limited access to healthcare, low educational attainment, and inadequate prenatal care (10). As such, MS is a critical marker for public health surveillance, given that timely diagnosis and appropriate treatment can effectively prevent CS. To mitigate these outcomes, the World Health Organization (WHO) recommends early syphilis screening during pregnancy as a key strategy for CS prevention. The disease is easily diagnosed through serological testing, underscoring the importance of early detection and adequate prenatal management in controlling congenital transmission (4, 11). In Brazil, syphilis is part of the Ministry of Health's official prenatal care protocols, which recommend routine screening during pregnancy. Rapid diagnostic tests for syphilis are available at primary care units. Programs like *Rede Cegonha* and the national strategy for the elimination of congenital syphilis aim to expand access to testing, enabling early detection, treatment, and improving maternal and child health. However, challenges in implementing these initiatives, especially in regions with greater socioeconomic vulnerability, may limit their reach and effectiveness.

Penicillin continues to be the first-line treatment for syphilis (2), which is readily available through Brazil's Unified Health System (SUS). MS is included on the Ministry of Health's National List of Notifiable Diseases, which mandates that cases be reported to health authorities via the Notifiable Diseases Information System (SINAN) in accordance with Consolidation Ordinance No. 4 of September 28, 2017. Previous research has shown that MS notification in Brazil is influenced by socioeconomic conditions and disparities in healthcare access, which can affect the accuracy and completeness of case reporting (10). Understanding the epidemiology of MS and its association with socioeconomic factors is essential for identifying key public health challenges and guiding policies and awareness campaigns aimed at reducing its incidence.

This study investigates the social vulnerabilities associated with MS in the Federal District (FD), Brazil. We analyzed the incidence patterns of MS and examined their correlation with the sociodemographic characteristics of the FD's Administrative Regions (ARs), using data from the Info Saúde DF portal (https://info.saude.df.gov.br/sifilissalasit/) and the most recent District Household Sample Survey—PDAD (PDAD, 2021). Socioeconomic factors such as income, access to healthcare, education, and housing conditions play a significant role in shaping women's vulnerability to infections. By integrating MS surveillance data with the social profiles of affected populations, this analysis provides valuable insights into the social and economic inequalities that influence maternal health outcomes. Addressing these disparities can help identify critical gaps in the healthcare system and guide the allocation of targeted resources, contributing to a more equitable approach to the prevention and management of STIs during pregnancy.

## 2 Material and methods

This is a retrospective ecological time series study based on publicly available secondary data. We examined the incidence of MS reported in the FD, Brazil, over a five-year period (2019–2023). The analyses were based on Info Saúde—DF portal data, managed by the FD Health Secretariat (SES-DF) (https://info.saude.df.gov.br) (12). This portal compiles data from Brazil's Notifiable Diseases System (SINAN) to provide specific information about the FD. SINAN is the national system managed by the Brazilian Ministry of Health that records and processes data on diseases and health conditions subject to compulsory notification.

For notification to SINAN, the diagnosis must be confirmed according to the clinical, epidemiological, and laboratory criteria established in the Clinical Protocol and Therapeutic Guidelines of the Brazilian Ministry of Health. In this study, the inclusion criteria comprised all notifications of MS cases between 2019 and 2023 among residents of the FD and individuals aged 10–54 years. Frequencies of incidence by year and AR were described. Data extraction took place between February and July 2024. No patients were involved in this study.

The FD comprises 33 administrative regions, including the capital city, Brasília, with notable heterogeneity in socioeconomic conditions. For this study, sociodemographic data were obtained from the PDAD conducted by the Distrito Federal Planning Company (Codeplan) in 2021 (13). According to Codeplan, the estimated FD population is 3,010,881 inhabitants, distributed across the ARs. To evaluate socioeconomic disparities, the 33 ARs were grouped into four income categories based on classifications from the 2021 PDAD, which draws on the Employment and Unemployment Survey of the FD. These groups are categorized as follows: high (Group I), medium-high (Group II), medium-low (Group III), and low (Group IV). The following sociodemographic indicators were collected: population size, educational attainment, age, race/color, and household income, which were included in the analysis (Supplementary Table S1).

All 33 ARs were included in the study, namely: Lago Sul, Park Way, Lago Norte, Sudoeste/Octogonal, Jardim Botânico, Plano Piloto, Águas Claras, Cruzeiro, Guará, Vicente Pires, Arniqueira, Sobradinho, SIA, Taguatinga, Candangolândia, Núcleo Bandeirante, Riacho Fundo, Gama, Ceilândia, Samambaia, Riacho Fundo II, Santa Maria, Sobradinho II, Brazlândia, Recanto das Emas, Planaltina, Varjão, Paranoá, São Sebastião, Itapoã, Sol Nascente, Fercal, and SIA/Estrutural.

Data from Info Saúde-DF and SINAN were organized in a Microsoft Office Excel 365 spreadsheet (Microsoft Corporation, Santa Rosa, CA, USA). The incidence of MS was extracted annually for each AR, along with the previously listed sociodemographic indicators. To determine the MS incidence, two approaches were adopted. First, we used the conventional method recommended by SINAN, in which the number of reported MS cases is divided by the number of live births and expressed per 1,000 live births. This calculation provides a direct measure of the epidemiological burden of MS. However, for the analyses involving sociodemographic indicators, rates were recalculated using the total AR population as the denominator and expressed per 10,000 inhabitants. This adjustment was necessary because sociodemographic data from PDAD are available by total population and not specifically for pregnant women or live births. While this alternative rate does not accurately represent the true epidemiological risk, as MS only affects pregnant women, it enables ecological comparisons with socioeconomic indicators across ARs.

To assess the association between MS incidence and sociodemographic indicators (educational attainment and per capita income), simple linear regression analysis was performed. Statistical significance was set at *p* < 0.05. Given the ecological design and the limited number of variables analyzed, these regression analyses are exploratory and do not adjust for potential confounding factors. Future studies employing multivariable regression models are recommended to elucidate better the complex relationships between social determinants and MS incidence. Quantitative data were summarized using means and standard deviations, where applicable. All analyses and data visualizations were done using GraphPad Prism® software (https://www.graphpad.com).

## 3 Results

This study analyzed the incidence of MS and its geographical distribution over a five-year period from 2019 to 2023 in the population of FD, Brazil. Using aggregated data from the Info Saúde DF portal and sociodemographic information from the 2021 District Household Sample Survey (PDAD) (CODEPLAN, 2022), we examined correlations between MS incidence and key socioeconomic and demographic variables across all 33 ARs of the FD. The comprehensive dataset allows for an exploratory assessment of spatial and temporal patterns in MS incidence within a diverse population context. A detailed summary of the extracted data, including demographic distributions and socioeconomic indicators, is presented in Supplementary Table S1.

**Figure 1:**
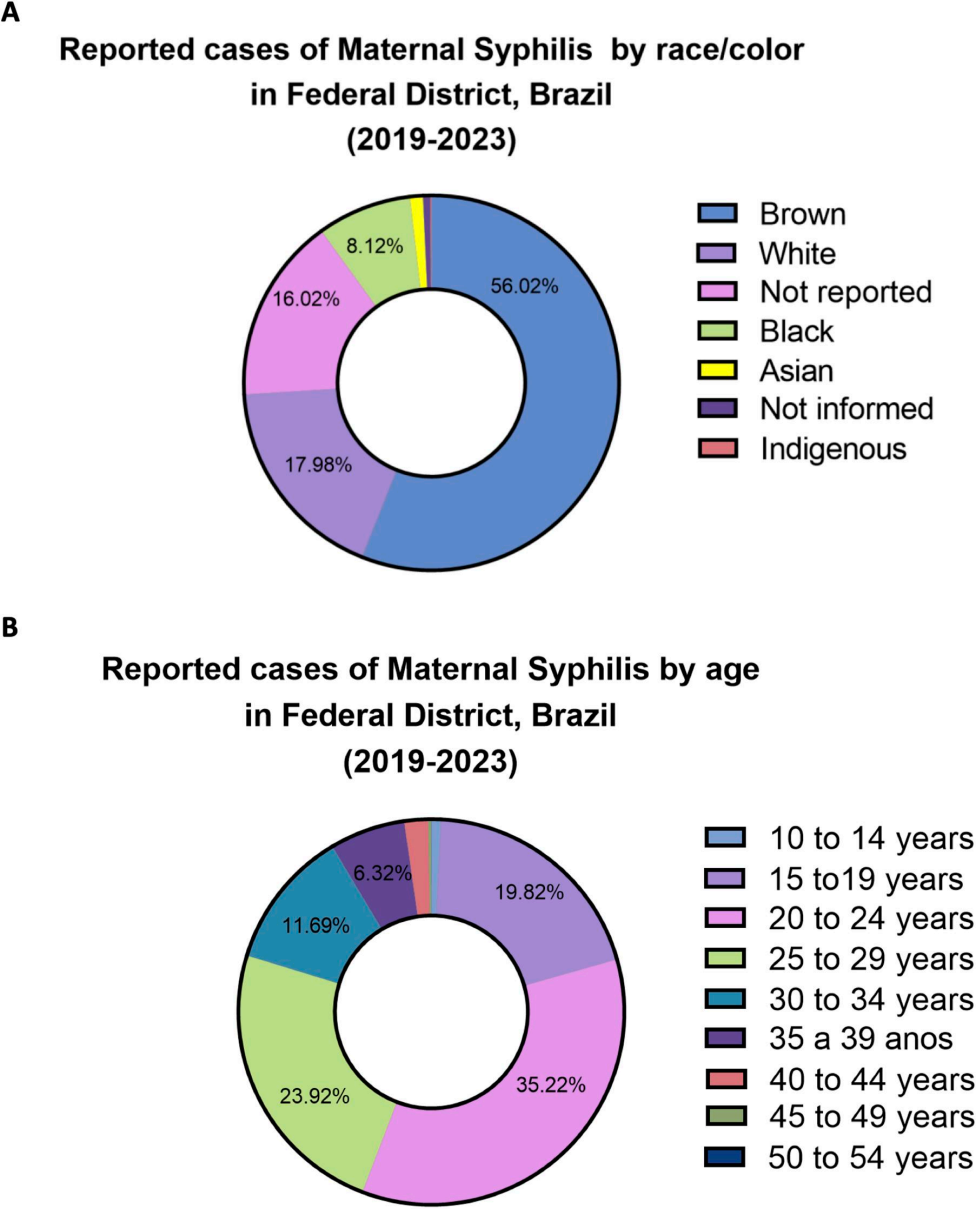
Sociodemographic distribution of reported maternal syphilis cases in the Federal District (FD), Brazil, from 2019 to 2023. **(A)** Distribution by self-declared race/color. Not reported: participants chose “ignored” as an answer option; Not informed: participants' answer to the question was not registered. **(B)** Distribution by age group.

The data revealed that from the 4,839 notified cases, the majority occurred among self-declared brown pregnant women, who accounted for 56% of notifications, followed by white women (17%), and those who did not report their race/color (16%). Black and yellow individuals represented 8% and 1% of cases, as shown in Figure 1A. Regarding age distribution, the age groups 15–19, 20–24, and 25–29 years together accounted for over 78% of reported MS cases during the study period, with the 20–24 age group representing the highest proportion, comprising 35% of all notifications (Figure 1B).

**Figure 2:**
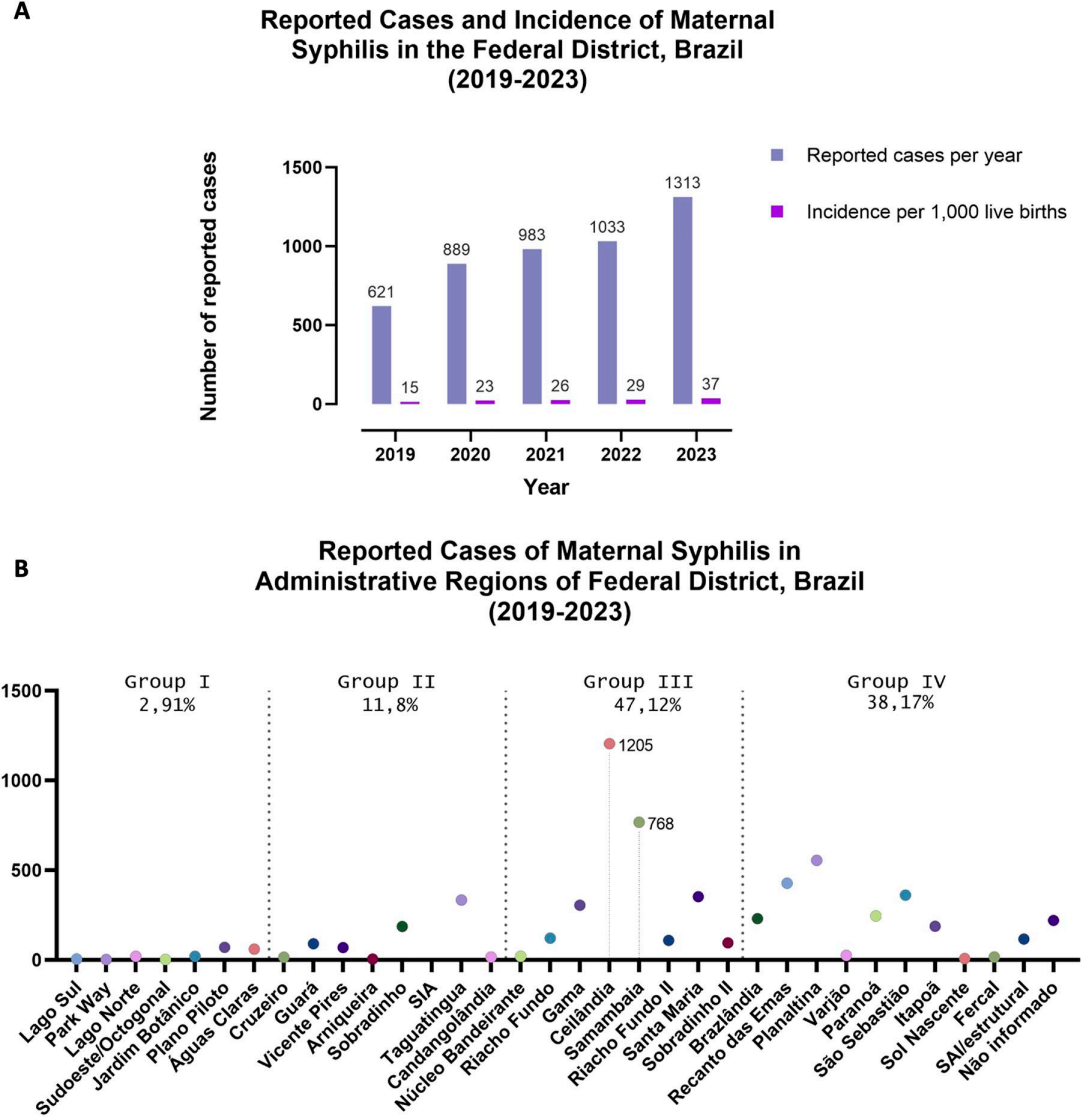
Reported cases of maternal syphilis in the Federal District (FD), Brazil, from 2019 to 2023. **(A)** Total number of reported cases per year and maternal syphilis cases per 1,000 live births. **(B)** Cumulative number of cases reported by Administrative Region (AR), with the percentage of cases indicated by income group, according to PDAD 2021 classification.

Data extracted from the SES-DF show that MS incidence has increased in recent years. In 2019, 621 cases of MS were reported, rising to 1,313 cases in 2023 (Figure 2A). The cumulative incidence of MS was 25.3 cases per 1,000 live births. The ARs with the highest absolute numbers of notification cases between 2019 and 2023 were Ceilândia and Samambaia, both classified under Group III. In contrast, Park Way and Sudoeste/Octogonal, belonging to Group I, recorded the lowest number of cases. Our analysis showed that MS notifications were predominantly concentrated in ARs classified in socioeconomic Groups III and IV, which together accounted for over 85% of all reported cases. Group III presented the highest average number of MS notifications (422 cases), followed by Group IV (217 cases). Groups II and I reported considerably lower averages, 82 and 26 cases, respectively (Figure 2B). These findings underscore the disproportionate burden of MS in socioeconomically disadvantaged regions.

**Figure 3:**
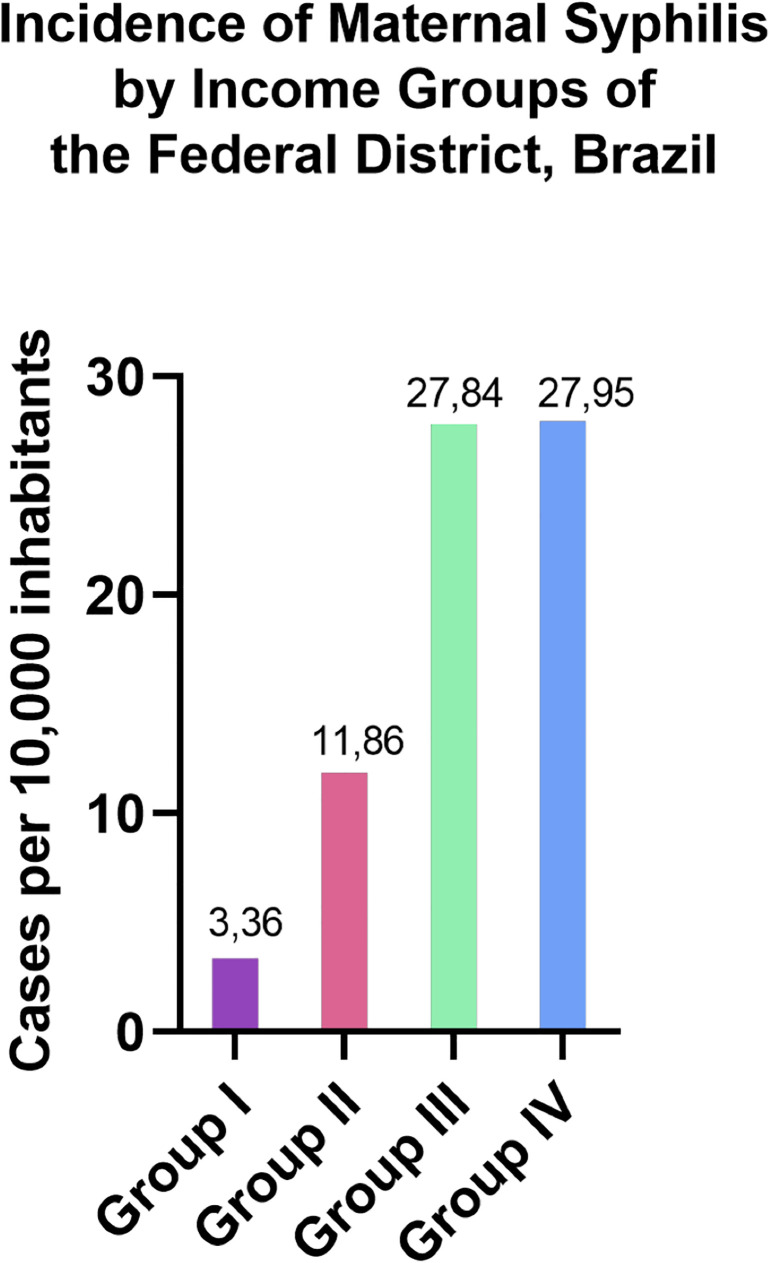
Reported cases of maternal syphilis (2019–2023) in the Federal District, Brazil, distributed according to population size across four income groups classified by the 2021 PDAD: high (Group I), medium-high (Group II), medium-low (Group III), and low (Group IV). These income groups reflect the socioeconomic stratification of the 33 Administrative Regions based on data from the FD's Employment and Unemployment Survey.

**Figure 4:**
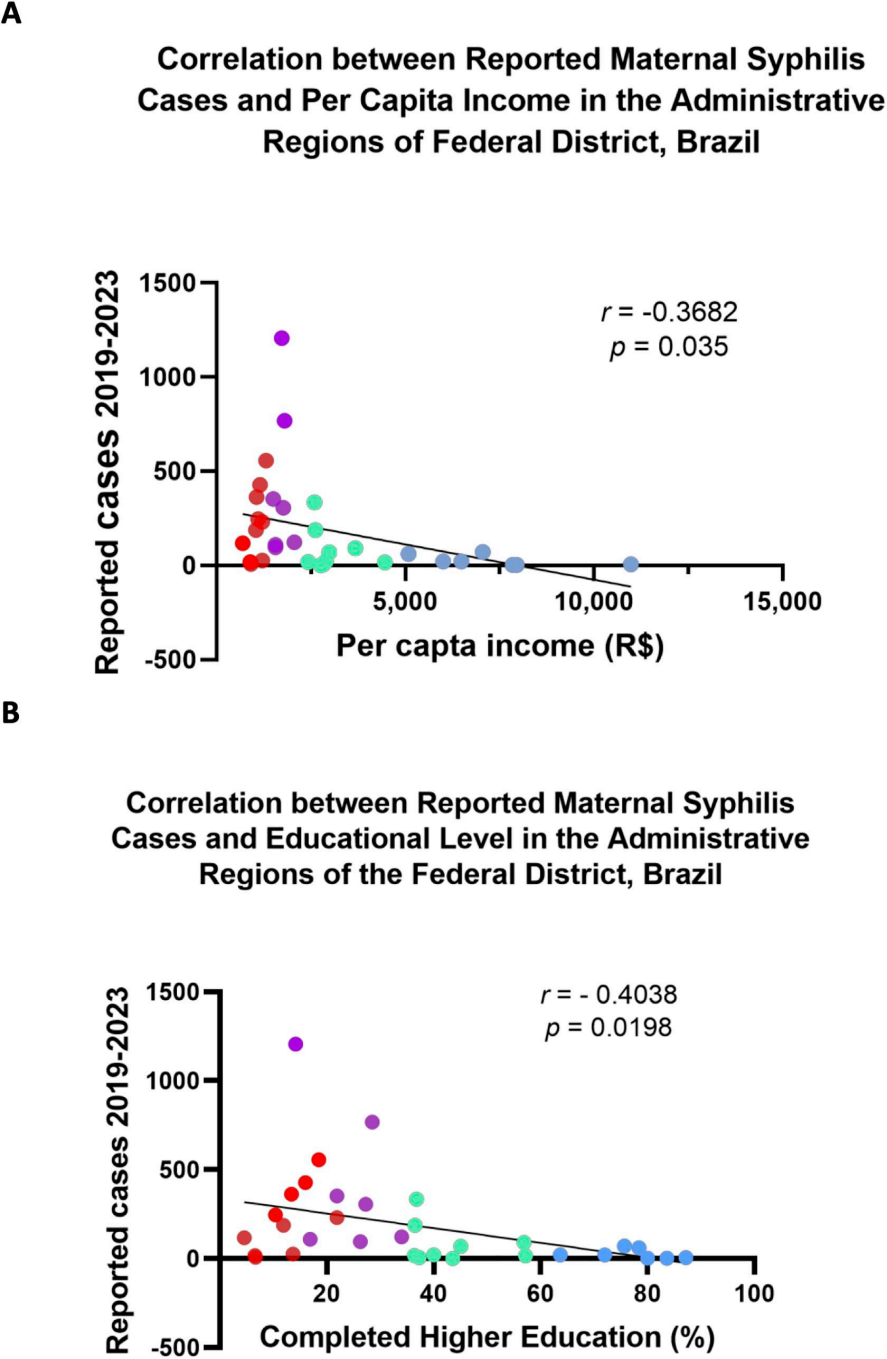
Correlation between reported cases of maternal syphilis and selected sociodemographic indicators across income-based groups of Administrative Regions (ARs) in the Federal District, Brazil (2019–2023). ARs were grouped into four income categories according to the 2021 PDAD classification: Group I (high income, blue), Group II (medium-high income, green), Group III (medium-low income, purple), and Group IV (low income, red). **(A)** Correlation with average household income. **(B)** Correlation of the proportion of residents with a complete higher education.

When adjusting for the average population size of each ARs, no significant difference was observed in the MS incidence rates between Groups III and IV, both presenting an average of 27 cases per 10,000 inhabitants. These were followed by Groups II and I, with average rates of 11 and 3 cases per 10,000 inhabitants, respectively (Figure 3). The incidence rates in Groups III and IV were significantly higher than in Groups II and I, reinforcing the association between MS burden and socioeconomic vulnerability. Additionally, the correlation analysis between MS case notifications and per capita income revealed a negative trend (*r* = −0.3682), which was statistically significant (*p* < 0.05) (Figure 4A). Although modest, this association suggests that lower-income regions report higher MS incidences, supporting the hypothesis that socioeconomic factors contribute to the spatial distribution of the disease in the FD.

Another correlation analysis was conducted to assess the relationship between the number of reported cases of MS in each AR and educational level, measured by the percentage of residents with completed higher education (Figure 4B). A moderate negative correlation was observed (*r* = −0.4038), indicating that higher education levels are associated with a lower incidence of MS. Groups III and IV, which reported the highest MS incidence, also exhibited the lowest percentage of residents with completed higher education, 24% and 12%, respectively, whereas Group I had an average of 77%.

## 4 Discussion

Currently, in Brazil and several other countries, syphilis is recognized as a re-emerging disease, as demonstrated by a significant rise in reported cases over recent years. In the FD, Brazil, surveillance data from 2019 to 2023 confirm this trend, showing a consistent increase in MS. The incidence rate rose from 15 to 37 cases per 1,000 live births, representing a 2.5-fold increase over the period. While improved surveillance and diagnostic efforts following the COVID-19 disruptions may have contributed to the 2023 data, the steady, pre-pandemic upward trajectory from 2019 suggests a sustained epidemic expansion that cannot be attributed solely to better reporting. Additionally, the observed predominance of MS notifications among self-declared brown individuals reflects broader structural inequalities in access to healthcare and information. According to Codeplan data, Group I ARs, such as Lago Sul (32.7%), Park Way (34.3%), Jardim Botânico (42.2%), Sudoeste/Octogonal (36.3%), and Plano Piloto (37.5%), have the lowest proportions of black and brown residents in the FD. These same regions also had the lowest MS incidence during the study period, suggesting a possible intersection between racial and socioeconomic vulnerability in the distribution of MS cases.

Analysis by age group further revealed that young women are particularly vulnerable to MS infection. This finding aligns with national epidemiological trends, which indicate a growing incidence of STIs among adolescents and young adults, who have been the primary contributors to the rising incidence of these diseases. According to the most recent HIV/AIDS Epidemiological Bulletin (14), 77,058 HIV cases were reported among men and 25,777 among women aged 15–24 between 2007 and 2022, making this age group the most affected during that period. Similarly, the 2023 Syphilis Epidemiological Bulletin (9) reported that from 2012 to 2023, most acquired syphilis cases were concentrated among individuals aged 20–29. An important additional finding was the steady and worrying increase in notifications among adolescents aged 13–19. Between 2012 and 2023, the number of reported cases in this group increased by more than three times among males and by five times among females, highlighting an alarming trend that indicates increased vulnerability and ongoing transmission in younger populations (9). These national patterns are mirrored in the FD, where over 78% of MS notifications occurred among individuals aged 15–29, with the 20–24 age group alone accounting for 35% of all reported cases. This highlights the urgent need for targeted health education, early screening, and expanded sexual and reproductive health services for young populations, particularly in socioeconomically vulnerable regions.

The data indicated that most MS notifications occurred in Groups III and IV, accounting for over 85% of cases. Among these Groups, Ceilândia and Samambaia presented the highest absolute numbers of MS notifications. While both are classified under Group III and share socioeconomic vulnerabilities and comparable health infrastructure with other ARs in the same group, these regions stand out as two of the most densely populated areas in the FD. This demographic factor may partially explain the elevated case numbers, as higher population densities can increase the total number of notifications.

This result suggests that lower-income populations may be more exposed to risk factors contributing to the high incidence of MS. Further analysis revealed a negative correlation trend between the number of MS cases and per capita income (*r* = −0.3682; *p* = 0.035), indicating that, on average, areas with higher income levels report fewer cases. This finding is consistent with the broader understanding that social vulnerability contributes to the burden of sexually transmitted infections. However, the modest strength of the correlation also suggests that income alone does not fully explain the spatial distribution of MS in the FD. It underscores the importance of considering other intersecting factors, such as access to prenatal care, educational attainment, and the quality of healthcare infrastructure.

We also explored whether the level of education correlates with MS incidence, with a particular focus on access to completed higher education. Our analysis revealed a negative correlation (*r* = −0.4038, *p* = 0.0198), indicating that higher levels of education are associated with fewer reported cases of MS. Groups III and IV, which exhibit the highest MS incidence, also demonstrate the lowest percentages of residents with completed higher education. This highlights the role of education in influencing the incidence of MS. Previous studies have shown that greater educational attainment increases awareness of STI risks and prevention strategies. Education encourages individuals to make informed decisions, adopt safer sexual practices, and effectively utilize healthcare services. Therefore, lower levels of education and limited access to information about STI prevention, such as condom use, may contribute to the higher incidence of MS in economically vulnerable populations.

The weak correlation between MS incidence and both per capita income and educational levels suggests that other factors may contribute to the rise in MS cases. Although modest, this correlation does not reduce the importance of the data, as the observed trends are real and quantifiable. One possible reason MS remains prevalent in socioeconomically vulnerable areas is that many pregnant women do not get prenatal care early or regularly. They may lack sufficient information about the importance of these check-ups or face other barriers that delay diagnosis and treatment. Although rapid syphilis tests are widely available through Brazil's official prenatal care protocols, challenges in reaching and engaging these populations might reduce the effectiveness of screening and intervention efforts. These likely include, but are not limited to, structural factors such as transportation difficulties, quality of local health units, and cultural barriers, alongside individual factors like health literacy.

This study adopted a simple linear regression approach to explore the relationship between MS incidence and key sociodemographic indicators in the FD, Brazil. While this method does not account for potential confounders, it is appropriate given the exploratory scope of the study and the limitations inherent to secondary data. The modest strength of the associations observed suggests that additional contributing factors may be involved, as the multivariable nature of the phenomenon cannot be ruled out. Future investigations using multivariate models, incorporating variables such as healthcare access, prenatal care coverage, and service quality, are necessary to gain a comprehensive understanding of the determinants of MS incidence in the FD and may help to clarify the complex interplay of the factors driving the rise in MS incidence in this region.

## 5 Conclusion

This study provides a preliminary spatial and temporal overview of MS incidence in the FD, Brazil, revealing that the ARs in socioeconomic Groups III and IV are the most affected. A modest but consistent negative correlation with income and education suggests greater vulnerability among young, brown, and less-educated women. Future studies could benefit from time-series or trend modeling approaches to better elucidate the complex determinants of MS. Public health policies aimed at reducing MS should prioritize lower-income populations, such as those residing in the ARs of Groups III and IV.

Understanding the distribution of MS within diverse populations can guide more effective and equitable public policies. Practical strategies to mitigate the incidence of MS and address health inequalities in vulnerable areas may include the implementation of locally adapted public health actions, such as strengthening surveillance and active case-finding in the most affected ARs. Other strategies may consist of establishing partnerships with schools and youth programs to enhance STI prevention; training healthcare teams in vulnerable regions for the early detection and appropriate management of syphilis; and developing accessible dashboards with MS indicators by AR to support local decision-making and accountability. Together, these efforts may contribute to reducing MS incidence, improving maternal and child health outcomes, and guiding more equitable public health responses across the FD, Brazil.

## Data Availability

The original contributions presented in the study are included in the article/Supplementary Material, further inquiries can be directed to the corresponding author.
