# Supplementary material for: Maternal syphilis in the Federal District, Brazil: a five-year analysis of notified data (2019–2023)
Source: Front Epidemiol. 2025 Nov 21;5:1613872. doi: 10.3389/fepid.2025.1613872 (PMC12678250; doi:10.3389/fepid.2025.1613872)
Supplement: Supplementary file 1 [file Table1.docx]

**Table 1:** Sociodemographic Profile of the Administrative Regions of the Federal District, Brazil. Extracted from PDAD, 2021.

| **ADMINISTRATIVE REGION (AR)** | **Income categories** | **GROUP** | **HOUSEHOLD INCOME (R$)** | **POPULATION SIZE** | **COMPLETE HIGHER EDUCATION (%)** |
| --- | --- | --- | --- | --- | --- |
| **Lago Sul** | High | I | 31.322,91 | 30.446 | 87,20 |
| **Park Way** | High | I | 18.138,22 | 23.081 | 80,05 |
| **Lago Norte** | High | I | 15.867,10 | 37.539 | 72,00 |
| **Sudoeste/Octogonal** | High | I | 15.390,62 | 55.366 | 83,63 |
| **Jardim Botânico** | High | I | 14.908,43 | 53.045 | 63,70 |
| **Plano Piloto** | High | I | 14.087,02 | 224.848 | 75,70 |
| **Águas Claras** | High | I | 14.056,73 | 120.107 | 78,40 |
| **Cruzeiro** | Medium-high | II | 9.858,61 | 30.860 | 57,20 |
| **Guará** | Medium-high | II | 7.978,96 | 142.083 | 56,90 |
| **Vicente Pires** | Medium-high | II | 7.775,30 | 78.561 | 45,10 |
| **Arniqueira** | Medium-high | II | 7.052,86 | 47.045 | 37,30 |
| **Sobradinho** | Medium-high | II | 6.010,84 | 73.438 | 36,50 |
| **SIA** | Medium-high | II | 5.998,42 | 1.737 | 43,60 |
| **Taguatingua** | Medium-high | II | 5.816,33 | 210.498 | 36,80 |
| **Candangolândia** | Medium-high | II | 5.612,47 | 16.339 | 36,40 |
| **Núcleo Bandeirante** | Medium-high | II | 5.486,03 | 24.093 | 40,00 |
| **Riacho Fundo** | Medium-low | III | 5.101,61 | 44.464 | 34,00 |
| **Gama** | Medium-low | III | 5.034,37 | 137.331 | 27,30 |
| **Ceilândia** | Medium-low | III | 4.491,07 | 350.347 | 14,20 |
| **Samambaia** | Medium-low | III | 4.128,25 | 247.629 | 28,50 |
| **Riacho Fundo II** | Medium-low | III | 3.863,50 | 72.988 | 16,90 |
| **Santa Maria** | Medium-low | III | 3.813,85 | 130.970 | 21,90 |
| **Sobradinho II** | Medium-low | III | 3.808,85 | 78.837 | 26,30 |
| **Brazlândia** | Low | IV | 3.425,65 | 55.879 | 21,90 |
| **Recanto das Emas** | Low | IV | 3.226,29 | 133.564 | 16,00 |
| **Planaltina** | Low | IV | 3.114,17 | 186.498 | 18,50 |
| **Varjão** | Low | IV | 2.907,20 | 8.953 | 13,70 |
| **Paranoá** | Low | IV | 2.843,63 | 69.858 | 10,40 |
| **São Sebastião** | Low | IV | 2.649,47 | 118.972 | 13,40 |
| **Itapoã** | Low | IV | 2.475,22 | 65.373 | 11,90 |
| **Sol Nascente** | Low | IV | 2.188,30 | 93.217 | 6,70 |
| **Fercal** | Low | IV | 2.186,12 | 9.388 | 6,60 |
| **SAI/estrutural** | Low | IV | 2.014,03 | 37.527 | 4,60 |
| **Total** |  |  |  | 3.010.881 |  |
